# A proposed synergetic mechanism for metal fume fever involving ZnO and Fe_3_O_4_ nanoparticles

**DOI:** 10.1038/s41598-022-19956-1

**Published:** 2022-09-19

**Authors:** Guillaume Suárez, Hélène Niculita-Hirzel, Daniela Correia, Jacques A. Pralong, David Vernez

**Affiliations:** 1 Department of Occupational and Environment Health, Center for Primary Care and Public Health, (Unisanté), University of Lausanne, rte de la Corniche 2, 1066 Epalinges-Lausanne, Switzerland; 2 Pulmonary Division, Department of Medicine, Geneva University Hospitals, 1211 Geneva, Switzerland

**Keywords:** Environmental chemistry, Risk factors, Photochemistry, Nanotoxicology

## Abstract

Metal fumes fever (MFF) is an inflammatory condition, whose mechanism is yet unclear, associated with the inhalation of metal fumes, particularly zinc. In this study we investigate experimentally the hypothesis of a two-step mechanism of MFF onset: (1) the photocatalytic production of airborne hydrogen peroxide (H_2_O_2_) via ZnO and (2) the production of hydroxyl radicals (HO**ׄ**) through Fenton reaction via magnetite (Fe_3_O_4_) nanoparticles. Photocatalysis and Fenton reaction products were measured using a multiscattering-enhanced absorbance device and assessing the degradation of bromophenol blue with microplate photometry, respectively. We observed that in the presence of UV, ZnO produces 3 to 4-times more H_2_O_2_ than UV alone or that non-UV irradiated ZnO. In the presence of biologically-relevant ligands, we also measured a Fenton reaction at physiological pH with either Fe(II), Fe(III) or Fe_3_O_4_ nanoparticles. Our results support the hypothesis of a two-step mechanism of MFF onset, in which the prior presence of Fe in the lungs exacerbates the oxidative stress, triggered by the photocatalysis of ZnO, a situation that could occurs when welding galvanized steel. More broadly, this raises the question of the role of the Fenton mechanism in respiratory exposure to metal particles and its possible contribution to other lung diseases.

## Introduction

Metal fume fever (MFF) is an occupational disease declared in welders and characterized by acute unspecific complaints such as influenza-like symptoms, fever, chills, headache, myalgias, malaise, cough and nausea^1^. The onset of symptoms typically occurs 4–10 h following the cessation of exposure to metal-containing fumes. The initial clinical findings are minimal, the respiratory rate, the pulmonary examination and the pulse oximetry being normal^2,3^. With continuous metal fume exposure over a week, tachyphylaxis is often seen, with the most severe symptoms after an exposure-free period (for example a week-end) and an improvement in symptoms over the course of the workweek. In a retrospective case review, the most frequent day of symptoms manifestation was Monday with a constant decrease in frequency during the following weekdays^1^. The initial chest X-ray is usually normal; laboratory studies are most of the time not necessary but may show a leukocytosis with leftward shift and an elevation of inflammatory markers. Pulmonary function test during the acute phase may demonstrate a decrease in vital capacity, which normalizes with recovery. The treatment of MFF is supportive and the prognosis good with a fast and total recovery over 12–48 h following exposure cessation in the majority of the cases^3^. Long-term sequels is very unusual. While severe cases of MFF are rare, benign cases are common. In 2006, the prevalence of MFF in the USA was estimated to 1500–2500 cases per year^2^.

Welding involves the generation of an electric arc as a powerful energy source able to make metals melt, with a broad emission spectral radiance covering the UV domain^4,5^. The production of fine/ultrafine metal oxide particles by the vaporization-condensation welding process is also well established. During stainless steel welding, metal vapours from the melt and electrode form, under cooling and oxidizing conditions, nanocrystals and fine particles of magnetite^6^. In the case of galvanized stainless steel, the generated aerosol is further enriched with ZnO particles. Despite the co-existence of high-energy radiation and metal oxide particles that can behave as semi-conductors, current mechanisms proposed for MFF do not consider UV and its possible interactions with metallic particles as causative variables but mainly focus on particles inhalation scenarios. The MFF was suggested to be induced by the inhalation of metallic particles such as emitted during welding. Indeed, the size of these particles—typically in the micro- and nanometric range—may readily reach the alveolar lung region. Exposure to concentrations as high as 77–600 mg Zn/m^3^ have been accepted to induce MFF^7^. Nevertheless, an exposure to 2 mg/m^3^ for 1 h has recently been shown to be sufficient to induce an increase in the level of hematologic markers^8^, while no hematologic or cardiovascular effects have been observed in subjects exposed to 0.5 mg/m^3^ of ZnO during 2 h^9^.

While the precise pathophysiology of MFF is still unknown, it is suspected that, once the welding fumes particles have been deposited in the bronchiolar and alveolar regions, the ZnO contained in induces the local formation of radical oxygen species (ROS). These ROS stimulate, via a cell-signalling pathway, the release of pro-inflammatory cytokines (including tumor necrosis factor (TNF), interlekin-6 (IL-6) and -8 (IL8)) by the pulmonary macrophages^11^ resulting in the development of symptoms such as those observed in MFF^10^. Epidemiological studies on welders have supported the time- and dose–response relationship between exposure and TNF, IL-6 and Il-8 concentration in bronchoalveolar lavage. However, while the release of pro-inflammatory cytokines was monitored in occupationally exposed population^11,12^, no studies were conducted, at our knowledge, on the ROS induction by the metal fumes.

In contrast, it was shown that exogenous ROS were generated^13^ by metal-containing particles issued from combustion sources such as vehicular traffic, incense burning, cigarette smoke or gas cooking (See, Wang^14^. In particular, a significant positive correlation has been found between Mn, V and Zn content of these particles and the ROS concentrations. Even further, water soluble metal fraction of ambient fine particles was associated with the ROS production^15^.

The nanometric nature of the particles is not sufficient to explain the occurrence of MFF in welders or its particular relationship with ZnO as recently demonstrated^8^. If we consider, on the one hand, the photocatalytic properties of ZnO and, on the other hand, the ability of transition metals to produce HOׄ via so-called Fenton redox reactions ^16^, other mechanisms can be considered.

Recently, Lenzen et al*.* reported an original oxidative stress scenario in which hydrogen peroxide acts as a pro-radical species able to distribute hydroxyl radical in cells via site-located Fenton-like reactions^17^. Similarly, we hypothesize here that environmental factors (UV, relative humidity) and the presence of ZnO and Fe_3_O_4_ nanoparticles enable the production of hydroxyl radicals—via the production of gaseous hydrogen peroxide—that contribute to exacerbating the inflammatory response to ZnO, thus explaining the highly circumstantial nature of the onset of MFF. Specifically, we want to assess whether a combination of UV-activated ZnO and Fe_3_O_4_ nanoparticles induces a photo-enabled oxidative stress pathway in epithelium cells, according to a two-step scenario:

- ZnO, in the form of steel coating layer or heat-produced nanoparticles, exposed to UV light first produces gaseous hydrogen peroxide through photocatalysis;
- Inhaled hydrogen peroxide triggers the in-situ generation of hydroxyl radicals via heterogeneous Fenton-like reaction with the Fe_3_O_4_ nanoparticles previously deposited in the lungs.

If established, these mechanisms would support the ROS-mechanism theory in the onset of MFF and could explain why it is frequently associated with galvanised steel welding, which involves UV light as well as Fe_3_O_4_ nanoparticles and ZnO layer.

## Results

**Figure 1:**
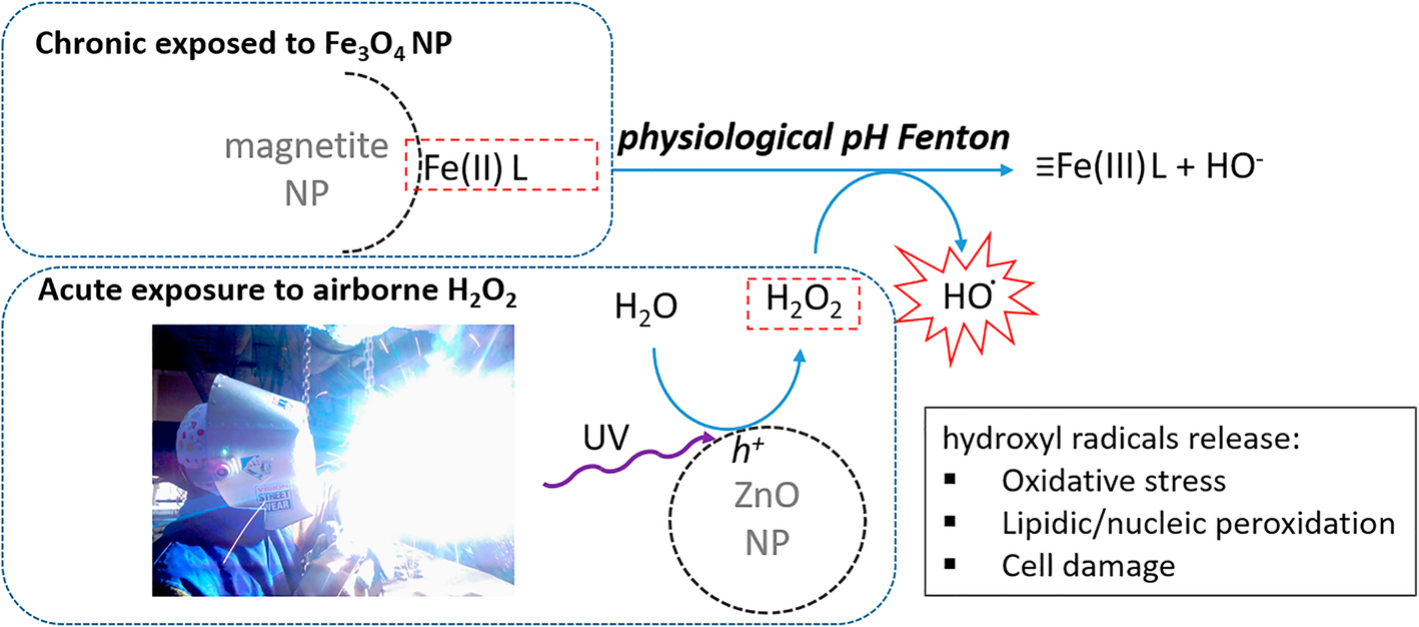
Schematics of the 2-steps MFF mechanism hypothesis that relies on specific conditions where chronic exposure to magnetite nanoparticles eventually stabilized with biological ligands in the airway undergoes Fenton reaction at physiological pH triggered by the acute exposure to gas-phase H_2_O_2_ generated via ZnO-UV photocatalysis.

The hypothesis of a two-step triggering mechanism of the MFF following exposure to welding fumes leans on a double assumption as schematized in Fig. 1: (1) during galvanized steel welding the ZnO layer—as well as the airborne heat-generated ZnO NP – undergoes UV-photocatalysis in the vicinity of the welding arc producing gas-phase H_2_O_2_; (2) When in contact with ligand-stabilized Fe_3_O_4_ NP previously deposited in the respiratory tract (acute/chronic exposure), inhaled H_2_O_2_ (acute exposure) triggers an in situ Fenton reaction with production of hydroxyl radicals.

### ZnO photocatalysis

**Figure 2:**
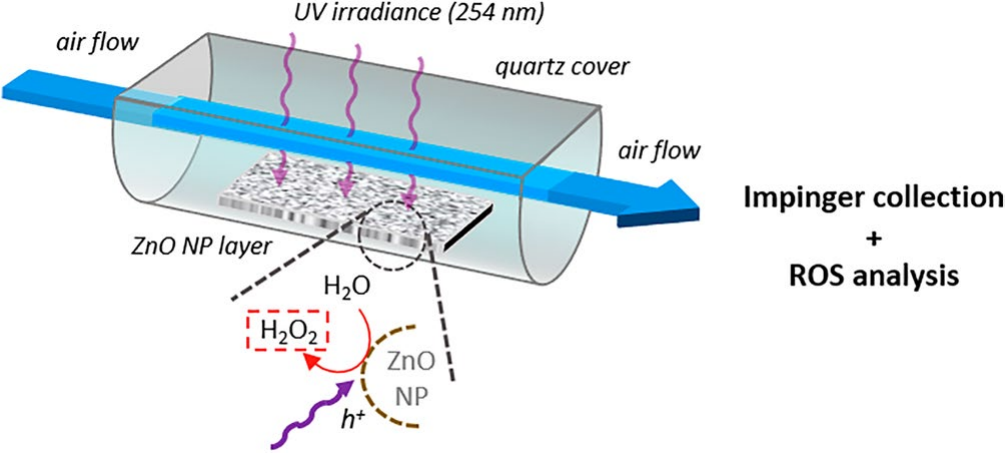
Schematic view of the experimental setup for ZnO nanoparticle-deposited layer exposure to UV radiation (254 nm) and subsequent quantitative determination of the airborne H_2_O_2_ photocatalytically produced.

**Figure 3:**
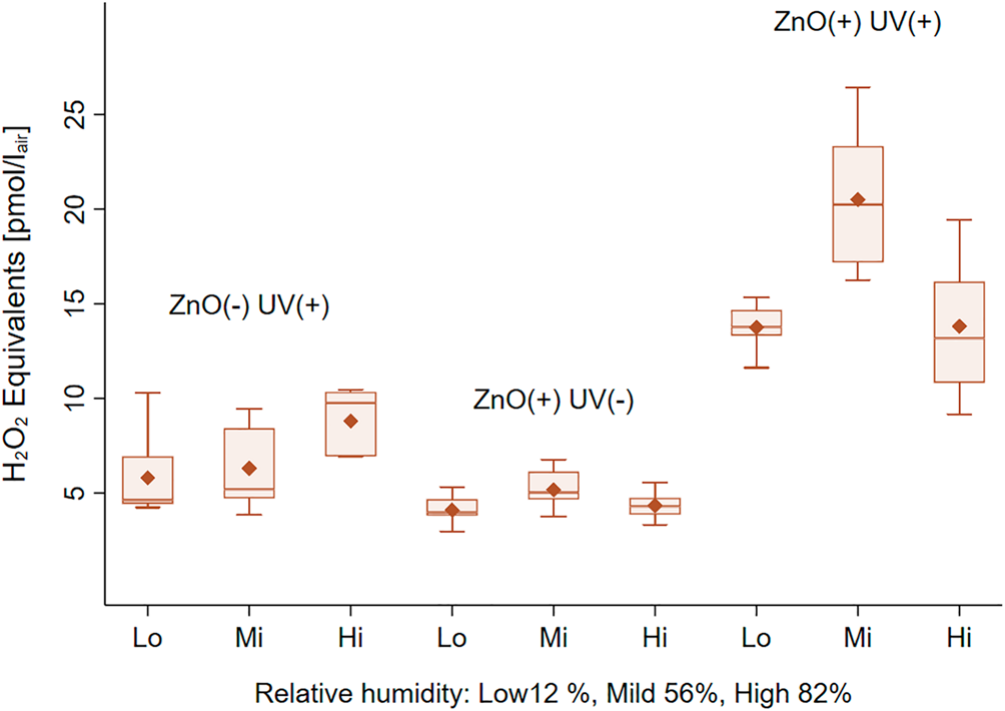
Oxidative potential of the airborne H_2_O_2_ (pmol/L_air_) photocatalytically produced after exposure to UV irradiance (254 nm), for three experimental conditions: UV alone, ZnO alone, UV and ZnO.

Focusing on the ZnO photocatalysis, the experimental setup represented in Fig. 2 aimed at mimicking the galvanized-steel welding scenario near the welded surface, where ZnO particles are exposed to UV light (254 nm) in the presence of air. In brief, the air stream (2L/min) leads the H_2_O_2_ photocatalytically-produced from the ZnO NP layer to the collection impinger filled with ultrapure water, for analysis. The results obtained at different relative humidity (RH) levels clearly show the effect of UV irradiance on ZnO-catalyzed generation of H_2_O_2_ (Fig. 3). Similarly to what was already observed for TiO_2_, the semi-conductor behavior of UV-irradiated ZnO enables fast oxidation of water molecules at the surface via ē/*h*^+^ formation^18^. Interestingly, the airborne H_2_O_2_ production is greater at mild RH conditions (56%)–12.3 pmol/L_air_—than at lower (12%) and higher (82%) RH ranges, both exhibiting a value of about 8.3 pmol/L_air_. The comparative analysis of the corresponding control experiments, ZnO/UV(-) and ZnO(-)/UV, highlights the formation of H_2_O_2_ originating from the cross-reactivity of UV-generation singlet oxygen ^·^O_1_ with water, the maximum air reactivity—5.1 pmol/L_air_—being reached at high RH. The calculation of the whole photocatalytic yields that encompass both ZnO and air contributions provides values comprised of 3.4, 4.0 and 3.2 corresponding respectively to low, mild and high RH conditions. In these experimental conditions, a maximum photocatalytic yield of 3.4 is achieved at mild RH specifically for ZnO, discarding the air-UV reactivity.

### Fenton reaction at physiological pH

Beside the photocatalytic generation of gas-phase H_2_O_2_, first assumption of our mechanistic hypothesis, the possibility of Fenton reaction to occur with Fe_3_O_4_ NP at physiological pH was studied. In addition, comparative experiments were carried out with Fe(II) and Fe(III) since the dissolution of Fe_3_O_4_ NP makes plausible the co-existence of Fe(II)/Fenton and Fe(III)/Fenton reactions. However, the zerovalent iron Fenton reaction (ZVI/Fenton) is not prone to happen since the condensation/oxidation of Fe(0) vapors predominantly gives rise to the formation of magnetite nanocrystals^6^.

**Figure 4:**
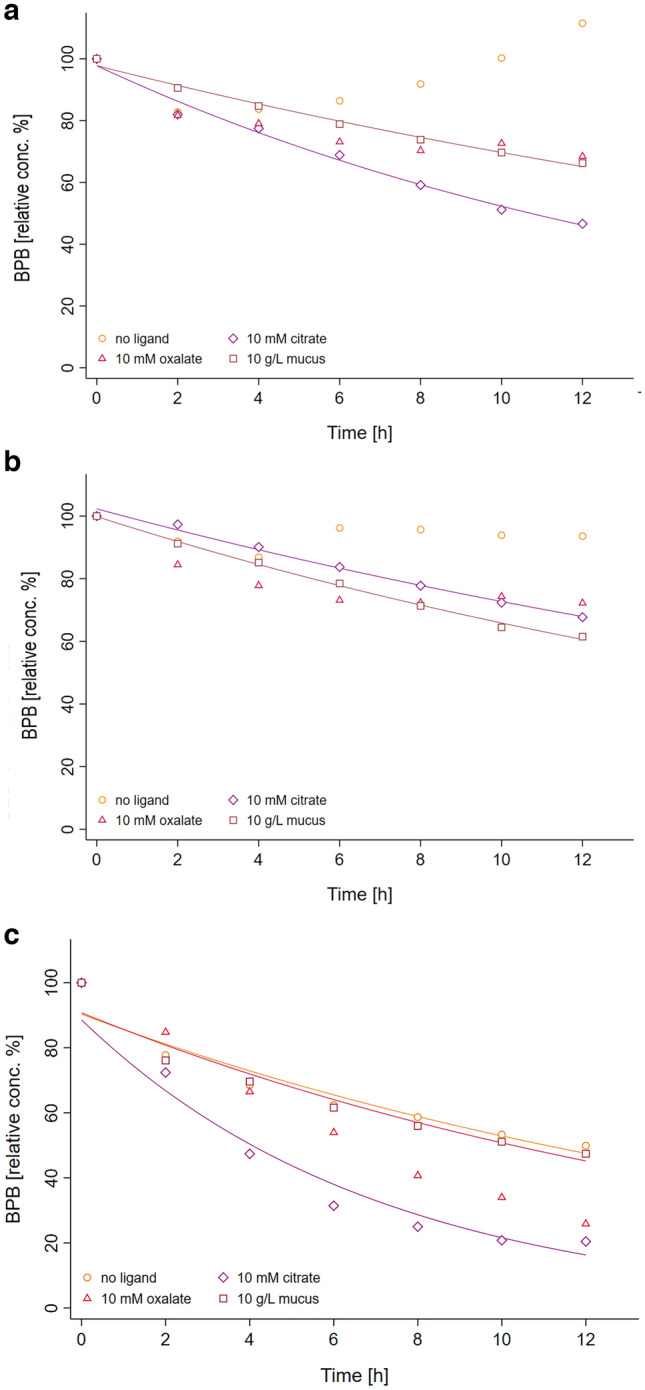
BPB degradation induced by the addition of H_2_O_2_ (5 mM) calculated from spectrophotometric kinetic data for Fe(II) (**a**), Fe(III) (**b**) and Fe_3_O_4_ NP (**c**). Each dot results from the correction of experimental absorbance values with the corresponding blanks (no BPB; no H_2_O_2_) and after conversion into BPB concentration.

Similarily to previous studies the degradation of an organic dye—here the bromophenol blue (BPB)-was used to indirectly assess the Fenton reaction through the generation of hydroxyl radicals in the system^19^. The kinetics of BPB degradation was followed using high throughput absorbance-based (590 nm) measurements in 96-wells microplate format. The curves in Fig. 4 show the BPB degradation kinetics obtained with either Fe(II), Fe(III) or Fe_3_O_4_ NP in the presence (or not) of biological ligands such as citrate, oxalate and diluted mucus. These kinetic curves are calculated from the control-corrected time response curves of the corresponding absorbance values. In the absence of ligand, no significant BPB degradation is observed for Fe(II) and Fe(III) after addition of H_2_O_2_, whereas the absorbance signal corresponding to Fe(II) slightly increases probably due to the progressive formation of an optical interference. In turn, the presence of citrate with both Fe(II) and Fe(III) induces a faster BPB degradation after 4 h indicating the generation of hydroxyl radicals in the system. Similarly, oxalate and mucus show to be favorable ligands of both iron ions to enable Fenton to happen at near-physiological pH, reaching relative values of [BPB] between 40 and 80% after 12 h. In contrast to Fe(II) and fe(III), Fe_3_O_4_ NP in the absence of ligand shows an effective BPB degradation as a consequence of H_2_O_2_ addition with about 50% of BPB being degraded over the period of measurement. The dye decolorization results further enhanced when citrate and oxalate act as chelating agents reaching 80% of degradation at maximum. The corresponding calculated apparent kinetic rate constants of the first-order, *k*_*1*_, summarized in Table 1, provide further information on the role of chelation in the enhancement of Fenton reaction under near-physiological conditions. The calculated rate constants enable both the quantitative comparison of the different ligands ability to drive Fenton reaction for a given individual iron species, Fe(II), Fe(III) or Fe_3_O_4_ NP as well as the comparative effect of a given ligand on Fe(II) and Fe(III) due to their experimental equimolar concentrations. However, providing that in the case of Fe_3_O_4_ NP the concentration of active Fe(II) and Fe(III) sites remains unknown, no cross-comparison might be established with the rate constants obtained for Fe(II) and Fe(III). Similar caution should be taken to interpret the effect of mucus in comparison to citrate and oxalate since its qualitative and quantitative composition is not precisely known. The analysis of the obtained apparent rate constants clearly indicates that citrate do act favorably on Fe(II), Fe(III) and Fe_3_O_4_ NP to either enable or improve the Fenton reaction at physiological pH. In turn, oxalate has no significant effect on Fe(II) and slightly improves the correlation coefficient of the first-order linearization for Fe(III) (R^2^ = 0.71) even though no apparent rate constant is to be consistently estimated from it. In the case of mucus, the improvement of the reaction’s kinetic from the no-ligand situation is visible for Fe(II) and to a greater extent for Fe(III). Native Fe_3_O_4_ NP do enable Fenton reaction in the presence of H_2_O_2_ and the Fenton reaction kinetic results further increased in the presence of citrate or oxalate, approximately by two-folds. A reverse effect is observed with Fe_3_O_4_ NP in the presence of mucus that seems acting either as an inhibitor for the Fenton reaction via scavenging of the hydroxyl radical eventually produced or a passivating coating through physisorption on the NP surface.

**Table 1:** Apparent kinetic rate constants of the first-order, (10^2^*k*_1_; min^−1^) and corresponding correlation coefficients (*R*^2^) calculated from the reaction kinetics of bromophenol blue degradation at neutral pH (when *R*^2^ < 0.9 the corresponding *k*_1_ is not calculated).

		No ligand	Citrate	Oxalate	Mucus
Fe(II)	*k*_*1*_	n.a	12.65	n.a	0.06
	*R*^*2*^	0.311	0.990	0.781	0.991
Fe(III)	*k*_*1*_	n.a	6.83	n.a	4.16
	*R*^*2*^	0.002	0.992	0.710	0.996
Fe_3_O_4_ NP	*k*_*1*_	5.42	12.25	11.43	0.10
	*R*^*2*^	0.939	0.911	0.997	0.954

## Discussion

Similarly to a few transition metal oxides that exhibit semi-conductor behavior—typically TiO_2_–ZnO strongly absorbs in the UV light domain with the formation of surface electron/hole pairs (energy band gap 3.36 eV)^20,21^. Although three crystal structures of ZnO–wurtzite, zinc blende and rocksalt—can be found, the former lattice is widely predominant (hexagonal structure with tetrahedral bond symmetry) due to its increased thermodynamic stability^22^. For both engineered ZnO NP and ZnO layer formed via O_2_ oxidation of galvanized steel (ZnO growth rate: 5 µm/year), the crystal lattice under ambient conditions is the Wurtzite structure^23–25^. In environmental conditions, the fast ē/h^+^ recombination occurs via two redox processes involving molecular oxygen and water. This results in the formation of a series of ROS such as superoxide radical anions, hydroxyl radicals and hydrogen peroxide. Providing the ultra-fast half-life time of hydroxyl radical (10^–6^ s)^17^ and the relative stability of gaseous hydrogen peroxide at ambient conditions (half-life time from 2 to 24 h) it is likely that the latter species contributes predominantly to the measurement. Hydrogen peroxide was found in the experimental airstream flowing from the UV-activated ZnO chamber with an average photocatalytic yield of about 4-folds at mild RH conditions. In a prior study focusing on the photocatalytic mechanism involved in UV-exposed TiO_2_ NP aerosol, we already observed that the main ROS response was originating from the gas phase^26^. The present results confirm, at least qualitatively, that part of the ZnO surface-produced hydrogen peroxide, as the most stable ROS, finds its way to the surrounding environment. The meaning of the optimal RH for photocatalysis to happen in our experimental setup remains unexplained, however it is clear that under realistic environmental conditions UV irradiance do produce airborne hydrogen peroxide. Shielding inert gas (argon or helium) are used in the context of professional welding precisely to prevent oxidation processes, therefore no ROS generation should be expected directly at the welding point. However, the gaseous hydrogen peroxide generation is prone to happen in a broader zone where the arc-induced UV radiations meet ambient air and ZnO, either in the form of airborne heat-generated nanoparticles or native metal-coating layer. Surprisingly, despite the known photocatalytic behavior of ZnO, the potential risk associated to ROS inhalation exposure during welding has been scarcely reported so far. In contrast, the generation of ozone via the UV-induced reaction of molecular oxygen into oxygen singlet is shown as a potential hazard in the welding-dedicated occupational safety literature. Far from pretending to closely mimic an occupational welding situation, although the estimated irradiance in the experimental setup (1.6 mW/cm^2^) is in the range of the occupational measured values (0.045–2.2 mW/cm^2^), the present experiments strongly indicate that galvanized-steel welding gives rise to an additional source of ROS such as hydrogen peroxide, able to freely diffuse from the ZnO UV-exposed surface to the surrounding space. The maximum H_2_O_2_ exposure concentration can be estimated, taking into account: (i) the total amount of H_2_O_2_ produced during one experiment (400 pmol in 10 min); (ii) the theoretical average volume inhaled during this period (75 L). Assuming that no dilution affects the concentration of H_2_O_2_ from the source to the breathing zone (worst case scenario), a resulting maximum H_2_O_2_ concentration is 5.3 nmol/m^3^. This value is much below the occupational exposure limit (OEL) of 21 µmol/m^3^ (0.71 mg/m3; 8 h TWA). On its own, H_2_O_2_ exposure originating from the photocatalytic behavior of ZnO in the presence of UV irradiance might hardly cause MFF symptoms, but the hypothesis of a triggering effect of the Fenton reaction is still plausible.

Regarding the second assumption of our hypothesis, the accelerating effect of chelating ligands on the Fenton and Fenton-like reaction rates at acidic pH in the presence of Fe(II), Fe(III) or Cu(I) is well reported in the literature as a promising approach for organic contaminant removal from wastewater^27,28^. Such reaction kinetics improvement is mostly attributed to the fact that the interaction metal-ligand lowers the redox potential E° of the complex by reducing its positive charge^29,30^. This impact of polydentate ligands on the complex is further reflected in the extension of the effective pH up to circumneutral values. In addition, iron chelation in the complex prevents the formation of Fe(III) hydroxides, enabling the Fe(III)/Fe(II) key cycle to occur^28^. Similar to the results here obtained, citrate belongs to the pool of biological chelating agents with mild reducing behavior which efficiently promote Fenton reaction from Fe(II) with the generation of hydroxyl radicals at near-neutral pH. However, the later species is not observed in the absence of ligand^28,31^. In turn, the lack of Fenton reaction observed with oxalate acting as chelating ligand might be attributed to the low stability constant of the complex formed with Fe(II), albeit oxalate/oxalic acid results is efficient to enhance photo-Fenton systems^30^.

The BPB degradation observed for native Fe_3_O_4_ at neutral pH translates the heterogeneous Fenton-like reaction occurring at the NP surface in the presence of H_2_O_2_. The unique redox properties of magnetite NP have been linked to its inverse spinel crystal structure in which half of the Fe(III) ions are tetrahedrally coordinated while the other half of Fe(III) and all the Fe(II) are octahedrally coordinated^32^. This crystal structure enables the mixed-valence iron oxide to undergo efficient electron transfer from Fe(II) and Fe(III) in the octahedral sites^33^. In other words, the two ion species constituent of magnetite can be reversibly oxidized and reduced without modifying the crystal structure^34^. The rational thinking in terms of redox potential leads to the assumption that the standard redox potential for ≡Fe(III)/≡Fe(II) at the Fe_3_O_4_ NP surface would necessarily be lower than the corresponding value for Fe(III)/Fe(II) in solution (E° = 0.77 V). In terms of mechanism, the efficient heterogeneous Fenton-like reaction observed with Fe_3_O_4_ NP at neutral pH, where the role of dissolved ions might be neglected, implies the formation of high-valent iron species such as ≡Fe(IV) as intermediates^33^. In addition, both the high surface/volume ratio and the location of active sites at the particle surface are responsible for the improved catalytic activity of the Fe_3_O_4_ NP in comparison to its bulk counterpart^34^. This heterogeneous Fenton-like reaction represents a well-established mineralization procedure to achieve reductive degradation of numerous organic pollutants via the generation of hydroxyl radicals^28,33,35–37^. Interestingly, Qu et al. recently showed that fast Fenton-like reaction is achieved in two-facet engineered BiOI membranes exhibiting electron rich/poor reaction sites^38^. By analogy to the facet-engineered membrane, the role of the spatial nanoconfinement of hydroxyl radicals at magnetite NP surface and the fast internal electron bridging from adjacent Fe(II)/Fe(III) sites are possible mechanisms that should be explored to explain the resulting Fenton efficiency. In this context of heterogeneous Fenton-like reaction, the observed positive action of citrate and oxalate on the reaction rate constant might be attributable to their mild reducing properties enabling efficient ≡Fe(III)/≡Fe(II) cycling. In contrast, the presence of mucus seems to hamper the accessibility of the surface reaction sites of the Fe_3_O_4_ NP resulting in a significant reduction of the corresponding Fenton-like reaction rate. One could hypothesize that the formation of protein corona on the Fe_3_O_4_ NP surface through physisorption or/and electrostatic interactions might cause such steric hindrance issue.

The results obtained in this study support the hypothesis of a two-step MFF triggering mechanism through a photocatalytic reaction of ZnO and the intra-pulmonary production of ROS species via a Fenton reaction. This experimental proof of concept, however, does not constitute a demonstration of the role of a Fenton mechanism in the inflammatory lung response. Although we have tried to reproduce experimentally conditions close to the lung environment in terms of pH, temperature (37 °C) and presence of ligands, these conditions are not sufficient to represent the complexity of the biological environment. Further studies, for example using 3D in vitro models, will still be needed to consolidate these hypotheses. Validation of mechanism would explain the prevalence of MFF in welders working on galvanized steel, which involves chronic exposure to iron nanoparticles and the circumstantial presence of ZnO surfaces and particles as well as UV light. Interestingly this mechanistic hypothesis also implies that chronic exposure to magnetite nanoparticles produces hydroxyl radicals in the cells of the respiratory tract via the Fenton reaction. The appearance of MFF would then be "only" the acute manifestation of oxidative stress, which is known to contribute to other pulmonary diseases over the long term. However, further studies under experimental conditions closer to those of welding must be carried out to consolidate this hypothesis.

## Materials and methods

### Reagents

ZnO (< 100 nm) and Fe_3_O_4_ magnetite (< 50 nm) nanopowders (Aldrich); ammonium iron(II) sulfate (Merk), chloride iron(III) hexahydrate (Merk) ; bromophenol blue (Aldrich); sulfuric acid 98% (Merck); xylenol orange tetrasodium salt (Fluka); D-sorbitol (Fluka) ; Sodium hydroxide (Fluka); hydrochloric acid 30% (VWR); sodium citrate tribasic dihydrate (Aldrich); oxalic acid (Aldrich), ultrapure water (MiliQ, 18.2 MΩ.cm at 25 °C, < 5 ppb total organic carbon, filtrated through 0.2 µm filter).

### Methods

#### Photocatalytic reactivity of ZnO nanoparticles (NP)

The experimental setup to assess the production of gaseous H_2_O_2_ from UV-induced ZnO photocatalysis is shown in Fig. 2. In brief, it consisted of a glass plate coated with ZnO NP (wet deposition of NP multilayer) inserted into an horizontal glass half-cylinder with air inlet/outlet plugs positioned at each extremity. Importantly, the half-cylinder was sealed with a quartz top plate facing a UV-C tube lamp (Camag model 29,200; 254 nm). Upstream, a two-paths system connected to pressurized clean air and water-filled nebulizer (Collison, 250 mL) enabled feeding the earlier described exposure chamber (2 L) with air at controlled relative humidity. Temperature and relative humidity monitoring was achieved using Data logger Ecolog TH1 (Elpro). During experiments, the air flow (1 Lmin^-1^) was driven through the exposure chamber in contact to the ZnO NP layer and finally directed out from it, towards a collection impinger (5 mL of miliQ H_2_O). Digital mass flow meters (red-y, Vögtlin Instruments AG, flow technology, Aesch BL, Switzerland) were used to measure air flows within the setup. At each run, the outcoming air was collected for 10 min and analyzed for H_2_O_2_ produced by photocatalysis. A direct reading particle counter (DustTrackII; National Instruments) was used to monitor the absence of particles in the vehicle air stream. Control experiments consisted of measuring the generated H_2_O_2_ i) without UV irradiance and ii) without ZnO NP.

Gaseous H_2_O_2_ concentrations collected via impinging were analyzed with the photonic device based on multiscattering-enhanced absorbance adapted to FOX-II assay^39^. FOX assay solution was daily prepared by mixing ammonium iron(II) sulfate (260 µM), xylenol orange (130 µM) and D-sorbitol (100 mM) into sulfuric acid (25 mM). The solution was kept in a coloured glass flask (100 mL). Immediately after each experimental run the collection solution (300 µL) was added into the FOX vial (700 µL) and the measurement processed for 3 min. Each run was measured in triplicate.

#### Effect of ligands on Fenton reaction

Ferric and ferrous solutions were prepared at 1 mM in MiliQ water; for the mixture iron-ligand solutions, 10 mM of citrate or oxalate were added. Mucus was collected from twelve MucilAir bronchial inserts—the in vitro cell model of the human airway epithelium cultured at the air liquid interface of Epithelix (Geneva, Switzerland) by centrifugation at 300 rpm for 5 min. Once pooled, the mucus was diluted in MilliQ water to reach a concentration of 10 g L^−1^ of iron in solution. For the preparation of magnetite NP suspensions, 150 mg of nanopowder was directly added into each of the ligands’ solutions (100 mL of oxalate, citrate or mucus) at the concentrations mentioned and sonicated for 5 min. All the solutions/suspensions were prepared daily and kept at 4 °C. Prior to use the pH was adjusted to 7. Bromophenol blue (BPB) aqueous solution was prepared from concentrated MeOH stock solution at a final 20 µM concentration.

The efficiency of the Fenton reaction under different iron speciation conditions.

**Figure 5:**
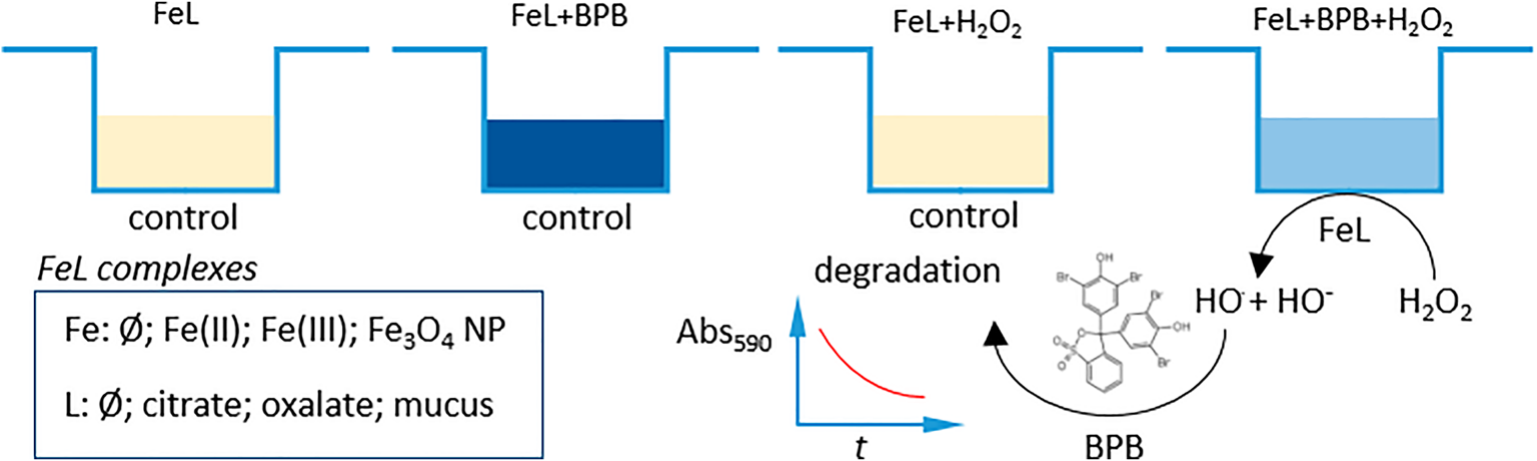
Experimental protocol for the quantitative determination of hydroxyl radical production from Fenton reaction with several iron/ligand systems at pH 7.0. The experiment is performed on 96-wells microplate with final reaction volumes of 200 µL/well. For each FeL system under study the combination of control and “full reaction” situations enables to determine the kinetic evolution of BPB degradation induced by radical hydroxyl release.

–Fe(II), Fe(III), Fe_3_O_4_ NP—and types of ligand–citrate, oxalate, mucus—was evaluated through the degradation of bromophenol blue (BPB) by the produced hydroxyl ions. BPB exhibits a strong molar absorptivity at 590 nm, thus its degradation due to Fenton reaction was monitored using a spectrophotometer (Infinite® 200 PRO multimode microplate reader from Tecan Group Ltd.) and microplate 96-wells system. Each well was filled with the iron-ligand solution/suspension (200 µL), BPB (10 µL of 20 µM) and H_2_O_2_ (10 µL of 100 mM). Two series of control experiments were carried out for each iron-ligand couple: (i) replacing H_2_O_2_ with ultrapure H_2_O; (ii) replacing BPB with H_2_O. Another control experiment consisted of replacing iron with H_2_O adjusted at pH 7.0, as stated in Fig. 5. The kinetics of BPB degradation were determined over 24 h (1 run/h; 37 °C; 3 s shaking between runs) with all the experimental conditions in triplicate, and analyzed on the same microplate. The BPB degradation profiles were calculated from absorbance values as follows:

$$BPB\ relative\ conc.\ \left(\%\right)=100*\frac{[BP{B}_{H2O2}-BPB{]}_{t}}{[BP{B}_{H2O2}-BPB{]}_{to}}$$

where: $$\left[BP{B}_{H2O2}\right]=[\left(FeL+BPB+{H}_{2}{O}_{2}\right)-\left(FeL+{H}_{2}{O}_{2}\right)]$$, and$$\left[BPB\right]=[\left(FeL+BPB\right)-\left(FeL\right)]$$.

#### Calculation of the apparent kinetic rate constants

In order to estimate the kinetic constants of Fenton reaction indirectly measured as BPB degradation, the corresponding absorbance data were converted into [BPB]—expressed in mol/L—via a calibration curve established at pH 7.0 in the range of 0–2 µM. For each of the iron/ligand situation the zero-, first- and second-order constants were determined from the adequate linearization plots [Santana_2019]. The greatest correlation coefficients were observed for the first-order model–*Ln([BPB])* = *f(t)*—providing significant apparent rate constant values, *k*_*1*_, whenever R^2^ > 0.9.

### Ethical approval

Ethical approval was not required for this study because no research was conducted on human or animal subjects.

## Data Availability

The experimental data generated during the current study are available from the corresponding author on reasonable request.
